# Immunogenicity and Safety of a Measles-Mumps-Rubella Vaccine Administered as a First Dose to Children Aged 12 to 15 Months: A Phase III, Randomized, Noninferiority, Lot-to-Lot Consistency Study

**DOI:** 10.1093/jpids/piz010

**Published:** 2019-03-08

**Authors:** Nicola P Klein, Remon Abu-Elyazeed, Michael Povey, Mercedes Macias Parra, Javier Diez-Domingo, Anitta Ahonen, Tiina Korhonen, Juan-Carlos Tinoco, Leonard Weiner, Gary S Marshall, Peter E Silas, Kwabena O Sarpong, Keith P Ramsey, John A Fling, David Speicher, Maribel Campos, Iona Munjal, Christopher Peltier, Timo Vesikari, Carmen Baccarini, Adrian Caplanusi, Paul Gillard, Stephane Carryn, Ouzama Henry

**Affiliations:** 1 Kaiser Permanente Vaccine Study Center, Oakland, California; 2 GlaxoSmithKline, Philadelphia, Pennsylvania; 3 GlaxoSmithKline, Wavre, Belgium; 4 Department of Infectious Diseases, Instituto Nacional de Pediatría, Mexico City, Mexico; 5 Fundacion para el Fomento de la Investigacion Sanitaria y Biomedica (FISABIO-Public Health), Valencia, Spain; 6 Vaccine Research Center, University of Tampere, Finland; 7 Laboratorio de Microbiología, Hospital General de Durango, Mexico; 8 Department of Pediatrics, SUNY Upstate Medical University, Syracuse, New York; 9 Department of Pediatrics, University of Louisville School of Medicine, Kentucky; 10 Wee Care Pediatrics, Layton, Utah; 11 Sealy Center for Vaccine Development, University of Texas, Galveston; 12 Jordan Ridge Kids & Teens, West Jordan, Utah; 13 Department of Pediatrics, University of North Texas Health Science Centre, Fort Worth; 14 Pediatric Pulmonary Division, Rainbow Babies and Children’s Hospital, Cleveland, Ohio; 15 Puerto Rico Clinical and Translational Research Consortium, San Juan; 16 Department of Pediatrics, Children’s Hospital at Montefiore, Bronx, New York; 17 Department of Pediatrics, University of Cincinnati College of Medicine and Pediatric Associates of Mt. Carmel, Inc, Ohio; 18 GlaxoSmithKline, Rockville, Maryland

**Keywords:** immunogenicity, vaccine, safety

## Abstract

**Background:**

MMR II (M-M-R II [Merck & Co, Inc.]) is currently the only measles, mumps, and rubella (MMR) vaccine licensed in the United States. A second MMR vaccine would mitigate the potential risk of vaccine supply shortage or delay. In this study, we assessed the immunogenicity and safety of another MMR vaccine (MMR-RIT [Priorix, GlaxoSmithKline]) compared with those of the MMR II in 12- to 15-month-old children who received it as a first dose.

**Methods:**

In this phase III, observer-blinded, noninferiority, lot-to-lot consistency clinical trial (ClinicalTrials.gov identifier NCT01702428), 5003 healthy children were randomly assigned to receive 1 dose of MMR-RIT (1 of 3 production lots) or MMR II along with other age-recommended routine vaccines. We evaluated the immunogenicity of all vaccines in terms of antibody concentrations (by using an enzyme-linked immunosorbent assay or electrochemiluminescence assay) and/or seroresponse rates 43 days after vaccination. We also assessed the reactogenicity and safety of the vaccines.

**Results:**

Immunoresponses after vaccination with MMR-RIT were robust and noninferior to those after vaccination with the MMR II. Immunogenicity of the 3 production lots of MMR-RIT was consistent; more than 97% of the children had a seroresponse to MMR components. The coadministered vaccines elicited similar immunoresponses in the MMR-RIT and MMR II groups. Both MMR vaccines resulted in comparable reactogenicity profiles, and no safety concerns were detected.

**Conclusions:**

If licensed, the MMR-RIT could provide a valid option for the prevention of measles, mumps, and rubella in children in the United States and would reduce potential risks of a vaccine shortage.

## Teaser

When compared with the MMR II vaccine (M-M-R-II) currently available in the United States, a first dose of MMR-RIT (Priorix) given to 1-year-old children elicited robust immunoresponses and raised no safety concerns.

The only combined measles, mumps, and rubella (MMR) vaccine currently available for use in the United States is the MMR II (M-M-R II, Merck & Co, Inc.) [1]. Routine 2-dose MMR II vaccination has led to the successful elimination of endemic measles and rubella and a decrease in the number of mumps cases by >99% compared with those in the prevaccine era [2–4]. However, these diseases are still present in the population, as revealed by the increasing number of measles and mumps outbreaks in recent years [4, 5]. Having another licensed MMR vaccine available in the United States would decrease the public health risk associated with potential interruptions in the MMR II vaccine supply.

The MMR-RIT (Priorix, GlaxoSmithKline [GSK]) is a combined MMR vaccine first licensed in the 1990s outside the United States and is currently available in more than 100 countries, where it has an indication and recommended schedules similar to those of the MMR II [6]. Regulatory requirements in the United States are now different than those when MMR-RIT was licensed originally, and a full development program to support US licensure of MMR-RIT is underway.

As part of that program, we conducted a phase III study to evaluate the immunogenicity and safety of the MMR-RIT compared with those of the MMR II when it is given as a first dose to 12- to 15-month-old children. This study was also aimed to assess the consistency of 3 production lots of MMR-RIT and evaluate the immunoresponses to vaccines routinely coadministered according to the US schedule.

## METHODS

### Study Design and Participants

This study was a phase IIIa, observer-blinded, randomized, controlled, noninferiority, lot-to-lot consistency study (ClinicalTrials.gov NCT01702428) conducted between November 2012 and April 2015 at 92 centers in Estonia, Finland, Mexico, Spain, and the United States.

We randomly assigned children aged 12 to 15 months in a 3:1 ratio to receive 1 dose of either 1 of 3 production lots of MMR-RIT or 1 of 2 commercial lots of the control MMR II (Supplementary Figure 1). All children concomitantly received a first dose of hepatitis A vaccine (HAV [Havrix, GSK]) and varicella vaccine (VV [Varivax, GSK]), and children in the United States also received a fourth dose of the 13-valent pneumococcal conjugate vaccine (PCV13 [Prevnar 13, Pfizer, Inc.]). The study consisted of in-person visits on day 0 (D0) and D42 and a telephone call on D180. We administered vaccines on D0 and collected blood samples (5 mL per child and visit) to assess antibody responses on D0 and D42.

We conducted the study in accordance with the Declaration of Helsinki and International Conference on Harmonisation Good Clinical Practice guidelines. The study protocol was reviewed and approved by independent ethics review committees or institutional review boards as applicable. Parents or legally acceptable representatives provided written informed consent for the children before enrollment.

Children aged 12 to 15 months in stable health were eligible. We excluded children with a history of, known exposure to, or previous vaccination against measles, mumps, rubella, varicella, herpes zoster, and/or hepatitis A disease, a history of allergic disease or reactions, or acute disease (See "Other eligibility criteria" in the Supplementary Methods section of the Supplementary Material).

### Randomization and Blinding

We stratified the randomization algorithm according to country, and we used a minimization procedure to ensure the correct balance of treatment groups within each center. We performed the treatment allocation at each center using a central randomization system on the Internet.

The study was double blinded for the MMR-RIT lot-to-lot consistency evaluation and observer blinded for the comparison between the MMR-RIT and MMR II. Because of the potential color differences between reconstituted MMR-RIT and MMR II, the staff handling these vaccines were not involved in the assessment of study end points.

### Study Vaccines

The composition of the MMR vaccines used is shown in Supplementary Table 1. The formulations of HAV, VV, and PCV13 have been described previously [7–9]. The MMR vaccines were injected subcutaneously in the left triceps; VV, subcutaneously in the right triceps; HAV, intramuscularly in the right anterolateral thigh; and PCV13 (only to children in the United States), intramuscularly in the left anterolateral thigh.

### Immunogenicity Assessments

We measured immunoglobulin G (IgG) antibodies to measles, mumps, rubella, hepatitis A virus, and varicella-zoster virus (VZV) by using commercial enzyme-linked immunosorbent assay kits (See "Immunogenicity assessments" in the Supplementary Methods section of the Supplementary Material). We quantified IgG antibodies against *Streptococcus pneumoniae* serotypes 1, 3, 4, 5, 6A, 6B, 7F, 9V, 14, 18C, 19A, 19F, and 23F using an in-house electrochemiluminescence assay (GSK, Wavre, Belgium, validation data, unpublished data).

We defined seroresponse as D42 antibody concentrations ≥200 mIU/mL for anti-measles, ≥10 enzyme-linked immunosorbent assay units (EU)/mL for anti-mumps (as used in previous studies [10]), ≥10 IU/mL for anti-rubella, and ≥75 mIU/mL for anti-VZV in children who were seronegative on D0 (See "Immunogenicity assessments" in the Supplementary Methods section of the Supplementary Material for the seronegativity thresholds). Seroresponse to hepatitis A virus was defined as a D42 anti-hepatitis A virus antibody concentration ≥15 mIU/mL in children with an antibody concentration <15 mIU/mL on D0 or, otherwise, a ≥2-fold increase in antibody concentration on D42. To define seroresponses, we used antibody concentration thresholds for anti-mumps and anti-rubella antibodies that were accepted by the Food and Drug Administration (FDA) as end points that define a clinically meaningful change in antibody titer; for anti-VZV antibodies, the seroresponse threshold was accepted by the FDA as a threshold commonly used in previous studies [11]. The seroresponse threshold for measles was based on comparison with titers from World Health Organization International Reference II and III. In the context of a head-to-head vaccine trial designed to demonstrate noninferiority, the FDA considered these thresholds acceptable immunoresponse levels.

### Reactogenicity and Safety Assessments

We recorded the solicited local adverse events (AEs) of pain, redness, and swelling at the MMR injection site from D0 to D3 (Supplementary Figure 1). We assessed the solicited general AEs of drowsiness, loss of appetite, and irritability/fussiness from D0 to D14 and assessed fever (temperature, ≥38.0°C), rash, parotid/salivary gland swelling, and febrile convulsions or signs of meningeal irritation from D0 to D42.

We documented unsolicited AEs from D0 to D42 and AEs that prompted an emergency department visit, serious AEs (SAEs), and new-onset chronic disease (NOCD) (Supplementary Table 6) during the entire study period (D0–D180).

We graded the intensity of all solicited AEs from 0 to 3 (See Supplementary Figure 2 footnote for definitions of grade 3 AEs). We considered all solicited local AEs as causally related to vaccination, and the investigator assessed the causality of other AEs.

### Statistical Analyses

We planned to enroll 5000 children to ensure randomization of 1250 children to each MMR-RIT lot group and 625 children to each MMR II lot group. Assuming a 20% nonevaluable rate in the according-to-protocol (ATP) cohort for immunogenicity, we estimated that 4000 children (1000 in each MMR-RIT lot group and 500 in each MMR II lot group) would be evaluable. Data for the 2 MMR II lots were pooled for all analyses.

The study had 5 primary objectives. The first and second primary objectives were to demonstrate the consistency of 3 lots of MMR-RIT in terms of seroresponse rates (SRRs) (objective 1) and adjusted antibody geometric mean concentrations (GMCs) (objective 2) for measles, mumps, and rubella on D42 (success criteria, the 95% confidence interval [CI] for the lot difference in SRR was within the [−5%; 5%] pre-specified margin and the 95% CI for the GMC lot ratio was within the [0.67; 1.5] pre-specified margin for all 3 antigens). The third and fourth primary objectives were to demonstrate noninferiority of the MMR-RIT (pooled lots) over the MMR II in terms of SRRs (objective 3) and antibody GMCs (objective 4) for measles, mumps, and rubella on D42 (success criteria, the lower limit [LL] of the 95% CI for the group difference in SRRs [pooled MMR-RIT − MMR II] was −5% or higher and the LL of the 95% CI for the adjusted GMC ratio [pooled MMR-RIT/MMR II] was ≥0.67 for all 3 antigens). The fifth primary objective was to demonstrate an acceptable immunoresponse to the MMR-RIT in terms of the SRR to measles, mumps, and rubella on D42 (success criterion, the LL of the 95% CI for the SRR in the pooled MMR-RIT group was ≥90% for all 3 antigens).

The secondary objectives of this study included assessment of the immunogenicity of the coadministered vaccines HAV, VV, and PCV13 in terms of SRRs and/or GMCs and assessment of the safety and reactogenicity of the MMR-RIT and MMR II (Supplementary Tables 3–6 and Figure 2). All the immunogenicity objectives were statistically powered except for the assessment of HAV immunogenicity in terms of SRRs.

All MMR immunogenicity analyses were conducted on the ATP cohort for immunogenicity, which included children for whom prevaccination and postvaccination serology results for at least 1 of the MMR antigens were available, who had a prevaccination concentration below the assay cutoff for at least 1 of the MMR antigens, who did not meet any elimination criteria up to D42, and who complied with protocol-defined procedures. We tested immunogenicity of the coadministered vaccines in subsets of children in the ATP cohort for immunogenicity (See "Statistical analyses" in the Supplementary Methods section of the Supplementary Material).

For each of the 3 separate MMR-RIT lots, for the pooled MMR-RIT lots, and for the MMR II group, we summarized the antibody GMCs for anti-measles, anti-mumps, and anti-rubella on D42 with their 95% CIs and the SRRs (defined as the percentage of children who had a seroresponse to these antibodies on D42) with their exact 95% CIs. We then tabulated, for each antigen, the difference in SRRs between groups (pooled MMR-RIT − MMR II) with their standardized asymptotic 95% CIs and the adjusted GMC ratios between groups (pooled MMR-RIT/MMR II) with their 95% CIs. To assess consistency across the 3 MMR-RIT lots, we computed the differences in SRRs (eg, MMR-RIT lot 1 − MMR-RIT lot 2) with their standardized asymptotic 95% CIs.

To assess the immunoresponses of the coadministered vaccines, we tabulated the GMCs for antibodies against VZV, hepatitis A virus, and *S pneumoniae* serotypes 1, 3, 4, 5, 6A, 6B, 7F, 9V, 14, 18C, 19A, 19F, and 23F on D42 with their 95% CIs. We computed the SRRs to VZV and hepatitis A virus on D42 with their exact 95% CIs. To test the prespecified noninferiority criteria, we calculated the adjusted GMC ratios between groups (pooled MMR-RIT/MMR II) with their 95% CIs for all the coadministered vaccines and the difference in SRRs between groups (pooled MMR-RIT − MMR II), with their asymptotic standardized 95% CIs for anti-VZV.

The safety and reactogenicity analyses were conducted on the total vaccinated cohort, which included all children with ≥1 documented administration of either MMR-RIT or MMR II. We tabulated the number and percentage of children (with exact 95% CIs) reporting each of the safety and reactogenicity variables assessed.

Statistical analyses were performed using SAS 9.3 (SAS, Inc, Cary, North Carolina) on SAS Drug Development 4.3 (See "Statistical analyses" in the Supplementary Methods section of the Supplementary Material).

## RESULTS

### Study Participants and Demographic Characteristics

**Figure 1.**
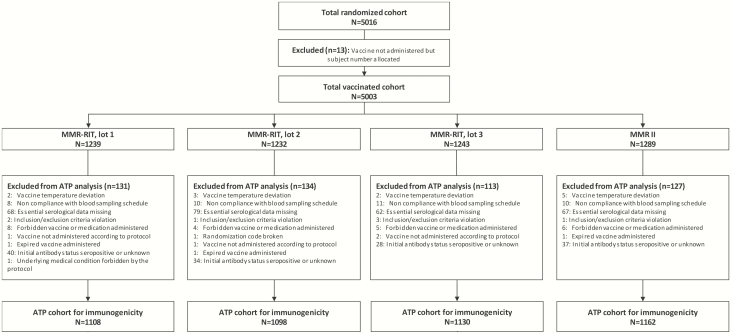
Flow diagram of the study participants. Abbreviations: ATP, according-to-protocol; N, number of children; n, number of children within the category.

We enrolled 5016 children, 5003 of whom were randomly assigned and received a single dose of MMR-RIT lot 1 (n = 1239), lot 2 (n = 1232), or lot 3 (n = 1243) or MMR II (n = 1289) (Figure 1). A total of 4759 children completed the study; the main reasons for discontinuation were loss to follow-up and consent of withdrawal not because of an AE. Overall, demographic characteristics were similar between the MMR-RIT and MMR II recipients (Table 1).

**Table 1.** Demographic Characteristics of the Study Participants (Total Vaccinated Cohort)

Characteristic	MMR-RIT Group (n = 3714)	MMR II Group (n = 1289)
Age at dose 1 (mean [SD]) (months)	12.3 (0.7)	12.3 (0.7)
Sex (n [%])^a^		
Female	1816 (48.9)	618 (47.9)
Geographic ancestry (n [%])		
White-Caucasian/European heritage	2814 (75.8)	970 (75.3)
African heritage/African American	169 (4.6)	70 (5.4)
Asian heritage	127 (3.4)	46 (3.6)
American Indian or Alaskan native	95 (2.6)	31 (2.4)
Other	509 (13.7)	172 (13.3)

Abbreviation: SD, standard deviation.

^a^Percentages shown are of children in the category.

### Immunogenicity Assessments

#### Noninferiority of the Immunoresponse to MMR-RIT Versus MMR II

On D42, the SRRs of the MMR-RIT group (pooled lots) were 98.2% for anti-measles, 98.4% for anti-mumps, and 97.3% for anti-rubella antibodies (Table 2). The SRRs to the MMR components observed after MMR-RIT vaccination were considered acceptable immunoresponses and were noninferior to SRRs in the MMR II group (criteria met; see “Statistical Analyses” for details) (Table 2).

**Table 2.** Noninferiority of MMR-RIT (Pooled Lots) Over MMR II in Terms of Anti-Measles, Anti-Mumps, and Anti-Rubella SRRs and Adjusted GMCs and Acceptable Immunoresponse of MMR-RIT (Pooled Lots) on Day 42 (According-to-Protocol Cohort for Immunogenicity)^a^

Parameter	MMR-RIT^b^	MMR II	Difference in SRRs (Pooled MMR-RIT SRR − MMR II SRR)^c^ or Adjusted GMC Ratio (Pooled MMR-RIT GMC/MMR II GMC)^d^
SRR (% [95% CI])			
Anti-measles	98.2 (**97.6** to 98.6)	98.0 (97.0 to 98.7)	0.18 (−**0.68** to **1.25**)
Anti-mumps	98.4 (**97.9** to 98.8)	97.6 (96.5 to 98.4)	0.81 (−**0.10** to **1.96**)
Anti-rubella	97.3 (**96.7** to 97.9)	98.5 (97.6 to 99.1)	−1.15 (−**2.00** to −**0.15**)
Adjusted GMCs (95% CI)			
Anti-measles	3165.2	3215.4	0.98 (**0.93** to **1.05**)
Anti-mumps	76.4	73.0	1.05 (**0.99** to **1.11**)
Anti-rubella	52.5	60.0	0.87 (**0.83** to **0.92**)

Abbreviations: CI, confidence interval; SRR, seroresponse rate (defined as the percentage of initially seronegative participants with a concentration above seroresponse)

^a^The adjusted GMC is the geometric mean antibody concentration from an analysis of variance model on log-transformed concentrations with treatment group and country as factors; the threshold for each antibody was 200 mIU/mL for anti-measles, 10 EU/mL for anti-mumps, and 10 IU/mL for anti-rubella. The numbers of participants for whom both prevaccination and postvaccination results were available were 3248 (MMR-RIT) and 1137 (MMR II) for anti-measles, 3187 (MMR-RIT) and 1107 (MMR II) for anti-mumps, and 3245 (MMR-RIT) and 1135 (MMR II) for anti-rubella.

^b^Acceptable immunoresponse to MMR-RIT was demonstrated if the lower limit of the 95% CI for the SSR in the MMR-RIT group (pooled lots) was ≥90% for anti-measles, anti-mumps, and anti-rubella.

^c^Standardized asymptotic 95% CI.

^d^The 95% CI for the adjusted GMC ratio (analysis of variance model: adjustment for country—pooled variance with >2 groups). Values in bold indicate that noninferiority criteria were met: the lower limit of the 95% CI for the difference in SRRs (pooled MMR-RIT − MMR II) was −5% or higher, and the lower limit of the 95% CI for the adjusted GMC ratio (pooled MMR-RIT/MMR II) was ≥0.67.

The antibody GMCs to the MMR components were similar between MMR-RIT and MMR II groups (Table 2). MMR-RIT vaccination was noninferior to MMR II in terms of GMCs for anti-measles, anti-mumps, and anti-rubella antibodies (criterion met; see Statistical analyses for details).

#### Lot-to-Lot Consistency of MMR-RIT

Children in the 3 MMR-RIT lot groups had high SRRs; antibodies against measles ranged from 97.8% to 98.6%, against mumps from 98.0% to 98.6%, and against rubella from 97.1% to 97.7%. The 3 lots were consistent in terms of SRRs and GMCs to all MMR components and met prespecified criteria (Supplementary Table 2).

### Immunoresponses to the Coadministered Vaccines

We evaluated the immunogenicity of VV in 2120 children enrolled in the United States (VZV subset). In addition, we also analyzed the immunogenicity of HAV in 1081 of these 2120 children (HAV subset) and the immunogenicity of PCV13 in the remaining 1039 children (PCV13 subset).

On D42, ≥90.2% of the children had a seroresponse against the VV (Supplementary Table 3). The SRR to VV in the MMR-RIT group was noninferior to that in the MMR II group. Anti-VZV antibody concentrations were also similar between study groups, and anti-VZV GMCs, comparing the MMR-RIT and MMR II groups, were noninferior (Supplementary Table 3).

SRRs to HAV were 88.8% in the MMR-RIT group and 87.1% in the MMR II group (Supplementary Table 3). GMCs of anti-hepatitis A virus antibodies were similar between study groups, and antibody concentrations in the MMR-RIT group were noninferior to those in the MMR II group (Supplementary Table 3).

The antibody concentrations to the components of PCV13 were comparable between study groups, and GMCs in the MMR-RIT group were noninferior to those in the MMR II group (Supplementary Table 4).

### Reactogenicity and Safety

The frequencies of reported solicited local and general AEs were similar in children in the pooled MMR-RIT and MMR II groups. The most frequently reported solicited local AE in both groups was injection site pain (25.9% in the MMR-RIT group, 28.1% in the MMR II group), followed by redness (24.5% in the MMR-RIT group, 25.2% in the MMR II group) (Supplementary Figure 2). Irritability/fussiness was the most frequently reported solicited general AE (63.3% in the MMR-RIT group, 65.9% in the MMR II group) (Supplementary Figure 2).

**Figure 2.**
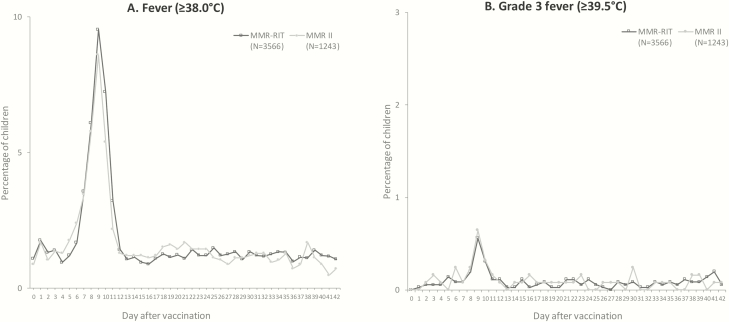
Prevalence of fever from day 0 to day 42 after vaccination (total vaccinated cohort). For visualization purposes, the scale of the y axis is different in each graph.

Reported fevers peaked during D5 to D12 (Figure 2) and were comparable between groups (19.7% in the MMR-RIT group, 18.2% in the MMR II group) (Supplementary Figure 2); ≤13.9% of the children had a fever considered causally related to vaccination, and ≤1.4% of the children had a grade 3 fever. The incidence of fever during D0 to D42 was 34.7% in the MMR-RIT group and 33.1% in the MMR II group; fever was considered causally related to vaccination in ≤18.9% of the children, and ≤2.9% of the children had a grade 3 fever. The daily prevalence and height of fever over the 43 days after vaccination were similar across the 3 groups of individual MMR-RIT lots and in the MMR II group (Figure 2).

Localized or generalized rashes were reported at the same frequency in both study groups (Supplementary Table 5). Measles/rubella-like rash occurred in ≤6.6% of the children, and varicella-like rash occurred in ≤7% of the children. From D0 to D42, 10 (0.3%) children from the MMR-RIT group and 3 (0.2%) children from the MMR II group had documented febrile convulsions or signs of meningeal irritation (Supplementary Table 5); 4 cases of febrile convulsions or signs of meningeal irritation in the MMR-RIT group and 2 in the MMR II group occurred during the D5 to D12 postvaccination period and were considered vaccination related. Medical advice was sought in 10 cases of febrile convulsions/signs of meningeal irritation. We did not record any episodes of parotid or salivary gland swelling in this study.

We found similar incidences of unsolicited AEs, SAEs, AEs that prompted an emergency department visit, and NOCDs between the MMR-RIT and MMR II groups (Supplementary Table 6). Grade 3 unsolicited AEs were reported in ≤6.6% of the children, and SAEs in ≤2.1%. NOCDs were documented in 3.4% of MMR-RIT recipients and in 3.7% of MMR II recipients; the most common NOCDs were atopic dermatitis (0.7% in the MMR-RIT group, 0.5% in the MMR II group) and eczema (0.4% in the MMR-RIT group, 0.8% in the MMR II group). No deaths occurred in this study.

## DISCUSSION

In this study, we found that vaccination with MMR-RIT induced robust immunoresponses that were noninferior to immunoresponses after MMR II vaccination in 12- to 15-month-old children. More than 97% of the children had a seroresponse against the 3 MMR-RIT components. We also found that the 3 lots of MMR-RIT were consistent in terms of SRRs and GMCs. Although the point estimates for the responses to the rubella component of MMR-RIT were slightly lower than those of the MMR II, the lower limit of the 95% CI was above the prespecified level, the GMC for rubella antibodies was fivefold higher than the threshold of 10 IU/mL, and the MMR-RIT rubella response met statistical noninferiority to MMR II. In addition, we found that the immunoresponses to the coadministered vaccines (VV, HAV, and PCV13) were similar between the two MMR vaccine groups (MMR-RIT and MMR II). Overall, these results show that the MMR-RIT met the prespecified criteria for noninferiority to the MMR II.

Our immunogenicity results are similar to those from previous first-dose studies of this vaccine with regard to seroresponses to the 3 MMR components [12, 13] and with SRRs and GMCs [14, 15].

The reactogenicity profiles of the 2 MMR vaccines were comparable, and we did not detect any new safety concerns. The peak in fever between D5 and D12 after vaccination was consistent with that in many previous reports [13, 16–19], and it has been attributed to the replication of the measles component of the MMR vaccines. The strains of measles virus in the MMR-RIT and MMR II are different; the MMR-RIT contains the Schwarz strain, and the MMR II contains the Edmonston-Enders (Moraten) strain. However, these strains are identical at the nucleotide level [20, 21], which is in line with the similar fever profiles observed in both of our vaccine groups. The mumps virus strain in the MMR-RIT is RIT 4385, derived from the Jeryl Lynn B strain used in the MMR II vaccine. The Jeryl Lynn component 1 is identical in both mumps strains at the protein level; only 1 silent mutation at the nucleotide level exists [22]. Accordingly, the reactogenicity profiles of both mumps strains are comparable, as shown in this and previous studies [23–25]. In addition, the MMR-RIT and MMR II contain the same rubella stain (Wistar RA27/3).

In this study, we found that approximatively 13% of the children experienced a rash related to vaccination, which was slightly higher than the incidence reported in other studies of MMR vaccines [13, 24, 25]. In this trial, parents and legally accepted representatives were instructed exhaustively on detecting and reporting different rash types, which could have prompted increased reporting. However, rashes related to vaccination were reported at similar rates for both vaccine groups.

A limitation of this study was that our results cannot be generalized to children who did not comply with the inclusion criteria but who would still be vaccinated in the clinical practice (eg, children with past exposure to 1 of the 3 viruses contained in the MMR vaccine).

In conclusion, results of this study show that immunoresponses after a first dose of MMR-RIT were noninferior to those of the US-registered vaccine MMR II. These results suggest that MMR-RIT could be a valid option for preventing measles, mumps, and rubella in children in the United States and helping to ensure that the United States has a second MMR vaccine available in the event of a hypothetical MMR vaccine shortage.

**Figure 3.**
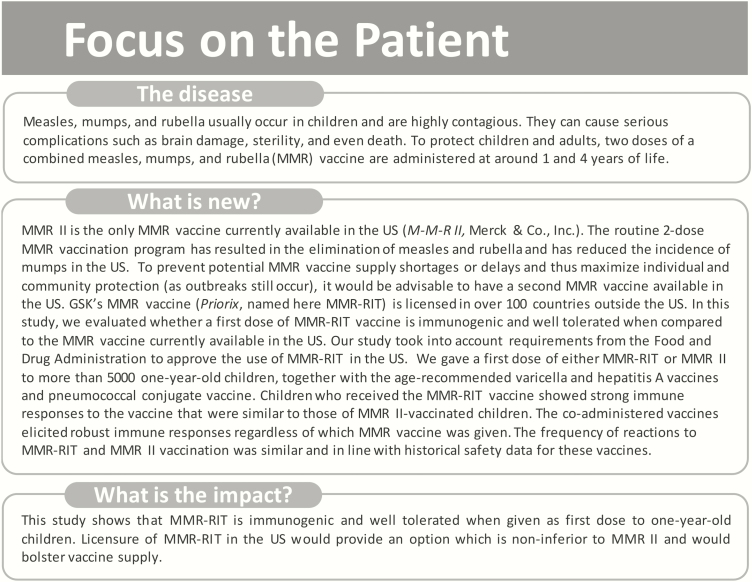
Focus on the patient. Summary contextualizing the outcomes of the study for the convenience of health care professionals.

For the convenience of health care professionals, a summary contextualizing the outcomes of this study is displayed in Figure 3.

## Supplementary Material

piz010_suppl_Supplementary_Materials

## Notes

***Acknowledgments.*** We thank the children who participated and their families and the investigators, nurses, and other study site personnel who contributed to this study. We thank Sara Rubio, PhD, and Urszula Miecielica, PhD (both XPE Pharma & Science, Wavre, Belgium, care of GSK), for providing medical writing services and Adrian Kremer, PhD (XPE Pharma & Science, care of GSK), for publication management.

***Financial support. *** This work was supported by GlaxoSmithKline Biologicals SA (GSK), which was the funding source and was involved in all stages of study conduct and analysis. GlaxoSmithKline Biologicals SA also took responsibility for all costs associated with the development and publishing of this article.

***Potential conflicts of interest. *** N. P. K., L. W., J. A. F., M. C., and C. P. report receiving grants from the GSK group of companies. N. P. K. also reports receiving grants from Merck & Co, Pfizer, Inc, Sanofi Pasteur, Novartis (now in the GSK group of companies), Protein Science (now Sanofi Pasteur), and MedImmune. L. W. also reports receiving grants from Merck & Co and Novartis and personal fees from Sanofi Pasteur for consulting services. M. C. also reports receiving a grant from the National Institute of Allergy and Infectious Diseases for clinical study. C. P. also reports receiving grants from Sanofi Pasteur and Regeneron. R. A.-E., M. P., P. G., S. C., and O. H. are employees of the GSK group of companies. R. A.-E., P. G., S. C., and O. H. hold shares in the GSK group of companies as part of their employee remuneration. M. M. P. reports receiving a grant, speaker’s honorarium, and consulting fee from Novartis and Sanofi Pasteur. J. D.-D. reports receiving grants, a speaker’s honorarium, and a consulting fee from the GSK group of companies, MSD, and SPMSD. G. S. M. is a scientific advisor and investigator for the GSK group of companies, Merck & Co, Novartis, Pfizer, Inc, Sanofi Pasteur, and Seqirus and reports receiving speaker’s honoraria from Pfizer, Inc, and Sanofi Pasteur. K. P. R. reports receiving research support from Novartis and research support from the GSK group of companies. I. M. served as a site investigator for the GSK group of companies and received funding as part of her involvement in the trial; she is an employee of Pfizer, Inc. T. V. declares receiving payment for lectures in the past from the GSK group of companies. C. B. and A. C. were employees of the GSK group of companies at the time the study was conducted. All other authors: No reported conflicts of interest. All authors have submitted the ICMJE Form for Disclosure of Potential Conflicts of Interest. Conflicts that the editors consider relevant to the content of the manuscript have been disclosed.

## Notes

Additional data: Anonymized individual participant data and study documents for further research can be requested from www.clinicalstudydatarequest.com.

Trademark statement: Priorix and Havrix are trademarks of the GSK group of companies. M-M-R II and Varivax are trademarks of Merck & Co, Inc. Prevnar 13/Prevenar 13 is a trademark of Wyeth LLC, marketed by Pfizer, Inc.
