# Supplementary material for: Immunogenicity and Safety of a Measles-Mumps-Rubella Vaccine Administered as a First Dose to Children Aged 12 to 15 Months: A Phase III, Randomized, Noninferiority, Lot-to-Lot Consistency Study
Source: J Pediatric Infect Dis Soc. 2019 Mar 8;9(2):194–201. doi: 10.1093/jpids/piz010 (PMC7192400; doi:10.1093/jpids/piz010)
Supplement: piz010_suppl_Supplementary_Materials [file piz010_suppl_supplementary_materials.docx]

## **Immunogenicity and safety of a measles-mumps-rubella vaccine administered as a first dose to children 12–15 months of age: a phase III, randomized, non-inferiority, lot-to-lot consistency study**

**Authors:** Nicola P Klein et al.

**Running title:** Immunogenicity and safety of MMR vaccine

**Corresponding author:**

Nicola P Klein

Kaiser Permanente Vaccine Study Center

1 Kaiser Plaza, 16th Floor

Oakland, CA 94612 USA

e-mail: [nicola.klein@kp.org](mailto:nicola.klein@kp.org)

**Supplementary Material**

**Supplementary Methods**

This study is registered in ClinicalTrials.gov (NCT01702428) and a summary of the study protocol is available at <https://www.gsk-clinicalstudyregister.com/study/115648?search=study&study_ids=115648#ps>.

Other eligibility criteria

We only enrolled children who were in stable health, as established by medical history and clinical exam, and for whom the investigator believed that their parent(s)/legally acceptable representative(s) could comply with protocol requirements. In the United States (US), we only included children who had previously received a 3‑dose primary series of a 13-valent pneumococcal conjugate vaccine (PCV13) with the last dose ≥60 days prior to study entry, and we excluded any child who had previously received a dose of 7-valent pneumococcal conjugate vaccine (*Prevnar/Prevenar*, Pfizer) or a fourth dose of any other pneumococcal conjugate vaccine.

Other exclusion criteria were:

- Child in care, i.e., a child placed under the control or protection of an agency, organization, institution or entity by the courts, the government, or a government body, acting in accordance with powers conferred on them by law or regulation. This includes children cared for by foster parents or living in a care home, but does not include children who are adopted or have been appointed a legally acceptable representative.
- Use of any investigational or non-registered product (drug or vaccine) other than the study vaccine(s) during the period starting 30 days before the day (D) of study vaccination (D0) or planned use during the entire study period.
- Concurrent participation in another clinical study, in which the child had been or would be exposed to an investigational or a non-investigational product (pharmaceutical product or device).
- Chronic administration (defined as 14 or more consecutive days) of immunosuppressants, or other immune-modifying drugs during the period starting 180 days prior to the study vaccination on D0 or any planned administration of immunosuppressive and immune-modifying drugs during the entire study.
  - For corticosteroids, this meant prednisone ≥0.5 mg/kg/day or equivalent.
  - Inhaled and topical steroids were allowed.
- (Planned) administration of a vaccine not foreseen by the study protocol during the period starting 30 days prior to the study vaccination on D0 and ending at the second visit (D42).
  - Inactivated influenza vaccine and monovalent *Haemophilus influenzae* type b conjugate vaccine could be given at any time, including the day of study vaccination. These vaccines had to be administered at a different location than the study vaccine(s).
  - Any other age-appropriate vaccine could be given starting at the second visit (D42) and anytime thereafter.
- Administration of immunoglobulins and/or any blood products during the period starting 180 days before the study vaccination on D0 or planned administration from the date of vaccination through the immunogenicity evaluation at the second visit (D42).
- History of measles, mumps, rubella, varicella/zoster, and/or hepatitis A disease.
- Known exposure to measles, mumps, rubella, and/or varicella/zoster during the period starting within 30 days prior to the first study vaccination.
- Previous vaccination against measles, mumps, rubella, hepatitis A, and/or varicella virus.
- Any confirmed or suspected immunosuppressive or immunodeficient condition, based on medical history and physical examination (no laboratory testing required).
- Blood dyscrasias, leukemia, lymphomas of any type, or other malignant neoplasms affecting the bone marrow or lymphatic systems.
- A family history of congenital or hereditary immunodeficiency.
- History of allergic disease or reactions likely to be exacerbated by any component of the vaccines, including hypersensitivity to neomycin, latex, or gelatin.
- Acute disease at the time of enrollment. Acute disease was defined as the presence of a moderate or severe illness with or without fever. Fever was defined as a temperature ≥38.0°C by any age-appropriate route. All vaccines could be administered to children with a minor illness such as diarrhea or mild upper respiratory infection without fever.
- Active untreated tuberculosis based on medical history.
- Any other condition which, in the opinion of the investigator, prevented the child from participating in the study.

Immunogenicity assessments

We measured antibodies to measles, rubella, and varicella-zoster virus (VZV) using a commercial enzyme-linked immunosorbent assay (ELISA) kit, *Enzygnost* (Dade Behring) at NÉOMED-LABS Inc., Quebec, Canada; antibodies against mumps using an ELISA kit at Pharmaceutical Product Development, Inc., Pennsylvania, US; antibodies against hepatitis A virus using an in-house ELISA (GSK, Belgium); and antibodies against *Streptococcus pneumoniae* serotypes 1, 3, 4, 5, 6A, 6B, 7F, 9V, 14, 18C, 19A, 19F, and 23F using an in-house electrochemiluminescence assay (GSK, Belgium). Of note, an electrochemiluminescence assay (and not an ELISA) was used to measure antibodies against *S. pneumoniae* in order to meet requirements from regulatory authorities.

We considered the following antibody concentrations at D0 to be seronegative: anti-measles, <150 mIU/mL; anti-mumps, <5 EU/mL; anti-rubella, <4 IU/mL; anti-VZV, <25 mIU/mL; and anti‑hepatitis A virus, <15 mIU/mL. No seronegativity threshold was defined for antibodies to *S. pneumoniae* because the analysis of post-vaccination samples for these antibodies did not take into account the pre-vaccination serostatus of the children. Seroresponse rates (SRRs) to *S. pneumoniae* components were not assessed as the PCV13 dose administered in the study was the fourth dose of the recommended schedule and high SRRs were expected.

Safety assessments

In the intensity scale from 0 to 3 used to assess the intensity of solicited adverse events (AEs), we defined grade 3 as: limb was spontaneously painful or crying when limb was moved (pain); redness or swelling of diameter >20 mm; AE preventing normal activity (drowsiness, irritability/fussiness, any rash, any febrile convulsions or signs of meningeal irritation, unsolicited AEs); crying inconsolably (irritability/fussiness); not eating at all (loss of appetite); temperature >39.5°C (fever); swelling with accompanying general symptoms (parotid/salivary gland swelling).

New onset chronic diseases were considered as AE of specific interest and included e.g., autoimmune disorders, asthma, type I diabetes, vasculitis, celiac disease, conditions associated with sub-acute or chronic thrombocytopenia, and allergies.

Statistical analyses

This study had five secondary objectives. The first secondary objective was to demonstrate non-inferiority of varicella vaccine (VV) in the MMR-RIT group compared with the MMR II group in terms of SRRs and geometric mean antibody concentrations (GMCs) for antibodies to VZV at D42, when VV was co-administered with MMR-RIT as compared to when VV was co-administered with MMR II. This was reached if the lower limit (LL) of the 95% confidence interval (CI) for the group difference in SRRs (pooled MMR-RIT minus MMR II) was ≥‑10%, and if the LL of the 95% CI for the adjusted GMC ratio (pooled MMR-RIT over MMR II) was ≥0.67.

Further secondary objectives were to demonstrate non-inferiority of hepatitis A vaccine (HAV) and PCV13 in the MMR-RIT group compared with the MMR II group in terms of GMCs for antibodies to hepatitis A virus at D42 (second secondary objective), and GMCs for antibodies to 13 serotypes of *S. pneumoniae* at D42 (third secondary objective), when HAV and PCV13 were co-administered with MMR-RIT as compared to when they were co-administered with MMR II. These two objectives were met if the LL of the 95% CI for the GMC ratios (MMR-RIT over MMR II) was ≥0.5 for each antigen tested.

The fourth secondary objective was to assess the immunogenicity of HAV in the MMR-RIT group compared with the MMR II group in terms of SRRs to anti-hepatitis A virus antibodies. The last secondary objective was to assess the safety and reactogenicity of MMR-RIT and MMR II when co-administered with HAV, VV (all children), and PCV13 (only children in the US) (objective 10).

All the immunogenicity objectives of the study (primary and secondary) were statistically powered except for the assessment of HAV immunogenicity in terms of SRRs. Each co-primary objective could only be reached if all the associated criteria were met and if all previous co-primary objectives were reached. Secondary objectives were assessed, with no hierarchy between them, only when all primary objectives had been met.

We tested immunogenicity of VV in approximately half of the children in the according-to-protocol cohort for immunogenicity, and defined this subset of children as VZV subset. We tested immunogenicity of HAV in approximately half the VZV subset (defined as HAV subset) and immunogenicity of PCV13 in the other half of the VZV subset (defined as PCV13 subset). All children in the VZV, HAV, and PCV13 subsets were enrolled in the US.

All analyses in this study excluded children with missing or non-evaluable measurements, which were not replaced. However, we replaced missing daily recordings of solicited AEs with grade 1 or with the maximum value recorded for the children, if any.

**Supplementary Tables and Figures**

**Supplementary Table 1.** Components of the MMR vaccines used in this study

| **Component** | **MMR-RIT** | **MMR II^a^** |
| --- | --- | --- |
| Measles virus | Schwarz strain, ≥10^3.0^ CCID_50_ | Edmonston Enders (Moraten) strain, ≥10^3.0^ TCID_50_ |
| Mumps virus | RIT 4385 strain, ≥10^4.3^ CCID_50_ | Jeryl Lynn strain, ≥10^4.1^ TCID_50_ |
| Rubella virus | Wistar RA 27/3 strain, ≥10^3.0^ CCID_50_ | Wistar RA 27/3 strain, ≥10^3.0^ TCID_50_ |
| Excipients | Anhydrous lactose  Sorbitol  Mannitol  Amino acids  Neomycin | Sorbitol  Sodium phosphate  Sucrose  Sodium chloride  Hydrolyzed gelatin  Recombinant human albumin  Fetal bovine serum  Neomycin  Other buffer and media ingredients |
| Diluent | Volume after reconstitution: 0.5 mL | |
| CCID_50_, cell culture infectious dose 50; TCID_50_, tissue culture infectious dose 50. ^a^From the *M-M-R-II* package insert: [www.fda.gov/downloads/BiologicsBloodVaccines/UCM123789.pdf](http://www.fda.gov/downloads/BiologicsBloodVaccines/UCM123789.pdf) | | |

**Supplementary Table 2.** Consistency between MMR-RIT lots in terms of anti-measles, anti-mumps, and anti-rubella seroresponse rates and geometric mean antibody concentrations at Day 42 (according-to-protocol cohort for immunogenicity).

| **Antibody** |  | | **Difference in SRR between lots (95% CI)^a^** | **Adjusted GMC ratio over lots (95% CI)^b^** | |
| --- | --- | --- | --- | --- | --- |
| Anti-measles | Lot 1 - Lot 2  Lot 1 - Lot 3  Lot 2 - Lot 3 | -0.54 (**-1.69**, **0.58**)  0.25 (**-0.98**, **1.50**)  0.79 (**-0.35**, **1.98**) | | | 0.99 (**0.91**, **1.06**)  0.97 (**0.90**, **1.05**)  0.99 (**0.91**, **1.06**) |
| Anti-mumps | Lot 1 - Lot 2  Lot 1 - Lot 3  Lot 2 - Lot 3 | 0.02 (**-1.05**, **1.09**)  0.63 (**-0.50**, **1.81**)  0.61 (**-0.53**, **1.79**) | | | 0.93 (**0.87**, **1.00**)  1.04 (**0.97**, **1.11**)  1.11 (**1.04**, **1.19**) |
| Anti-rubella | Lot 1 - Lot 2  Lot 1 - Lot 3  Lot 2 - Lot 3 | 0.14 (**-1.30**, **1.58**)  -0.49 (**-1.86**, **0.86**)  -0.62 (**-2.02**, **0.74**) | | | 1.08 (**1.01**, **1.15**)  1.00 (**0.94**, **1.07**)  0.93 (**0.87**, **0.99**) |

Adjusted GMC, geometric mean antibody concentration adjusted for country; CI, confidence interval; SRR, seroresponse rate defined as the percentage of initially seronegative participants with concentration above seroresponse threshold for each antibody (200 mIU/mL for anti-measles, 10 EU/mL for anti-mumps, and 10 IU/mL for anti-rubella).
^a^ Standardized asymptotic 95% confidence interval.
^b^ 95% confidence interval for the adjusted GMC ratio (analysis of variance [ANOVA] model: adjustment for country - pooled variance with more than 2 groups).
Values in bold indicate consistency criteria met: for each pair-wise comparison, 95% CI for the difference in SRR between lots had to be within the [-5%; 5%] margin, and 95% CI for the GMC ratio had to be within the [0.67; 1.5] margin.

**Supplementary Table 3.** Non-inferiority of MMR-RIT (pooled lots) compared with MMR II in terms of seroresponse rate and geometric mean antibody concentration to antibodies to the co-administered varicella vaccine and hepatitis A vaccine at Day 42 (according-to-protocol cohort for immunogenicity, VZV and HAV subsets).

|  |  | | **Difference in SRR (pooled MMR-RIT minus MMR** **II) % (95% CI)^a^** |
| --- | --- | --- | --- |
| **Antibody** | **MMR-RIT** | **MMR II** |  |
|  | **SRR % (95% CI)** | **SRR % (95% CI)** |  |
| anti-VZV | 92.2 (90.7, 93.5) | 90.9 (88.2, 93.2) | 1.30 (**-1.31**, 4.29) |
| anti-hepatitis A virus | 88.8 (86.3, 90.9) | 87.1 (82.5, 90.8) | N.A. |
| **Antibody** | **MMR-RIT** | **MMR II** | **Adjusted GMC ratio (pooled MMR-RIT over MMR** **II) % (95% CI)^b^** |
|  | **GMC (95% CI)** | **GMC (95% CI)** |  |
| anti-VZV | 169.6 (164.3, 175.0) | 167.2 (158.2, 176.7) | 1.01 (**0.95**, 1.08) |
| anti-hepatitis A virus | 41.8 (39.2, 44.7) | 42.8 (38.3, 47.9) | 0.98 (**0.86**, 1.11) |
| CI, confidence interval; GMC, geometric mean antibody concentration; N.A.: not assessed (not a study objective); SRR, seroresponse rate defined as the percentage of initially seronegative participants with antibody concentration ≥75 mIU/mL (for anti-VZV) or ≥15 mIU/mL (for anti-hepatitis A virus) at Day 42; VZV, varicella zoster virus. VZV subset: subset of children in the ATP cohort for immunogenicity for whom we tested immunogenicity of varicella vaccine; HAV subset: subset of children in the VZV subset for whom we tested immunogenicity of hepatitis A vaccine. See details in Supplementary material. Number of children: for anti-VZV, MMR-RIT=1492 and MMR II=540; for anti-hepatitis A virus, MMR‑RIT=748 and MMR II=271 (children from the VZV or HAV subset with available results and who were seronegative prior to vaccination). ^a^ Standardized asymptotic 95% confidence interval. ^b^ 95% confidence interval for the adjusted GMC ratio (analysis of variance [ANOVA] model). Values in bold indicate non-inferiority criteria met: lower limit of the 95% CI for the difference in anti-VZV SRRs (pooled MMR-RIT minus MMR II) ≥-10%; LL of the 95% CI for the anti-VZV adjusted GMC ratio (pooled MMR-RIT over MMR II) ≥0.67; and LL of the 95% CI for the anti-hepatitis A virus GMC ratios (MMR-RIT over MMR II) ≥0.5. | | | |

**Supplementary Table 4.** Non-inferiority of MMR-RIT (pooled lots) compared with MMR II in terms of geometric mean antibody concentrations to serotypes of the co-administered 13-valent pneumococcal conjugate vaccine at Day 42 (according-to-protocol cohort for immunogenicity, PCV13 subset)**.**

| **Antibody to *S. pneumoniae* serotype** | **MMR-RIT** | | | **MMR II** | | **Adjusted GMC ratio (MMR-RIT over MMR**  **II) % (95% CI)^a^** |  |
| --- | --- | --- | --- | --- | --- | --- | --- |
|  | **N** | **Adjusted GMC** | **N** | | **Adjusted GMC** |  |  |
| 1 | 740 | 2.26 | 256 | | 2.39 | 0.94 (**0.85**, 1.05) | |
| 3 | 739 | 0.50 | 255 | | 0.50 | 0.99 (**0.91**, 1.08) |  |
| 4 | 732 | 1.62 | 255 | | 1.84 | 0.88 (**0.79**, 0.98) |  |
| 5 | 738 | 2.09 | 256 | | 2.28 | 0.92 (**0.83**, 1.01) |  |
| 6A | 740 | 5.82 | 256 | | 5.76 | 1.01 (**0.92**, 1.11) |  |
| 6B | 739 | 5.81 | 256 | | 5.92 | 0.98 (**0.89**, 1.09) |  |
| 7F | 739 | 3.66 | 256 | | 3.89 | 0.94 (**0.86**, 1.03) |  |
| 9V | 740 | 2.30 | 256 | | 2.32 | 0.99 (**0.90**, 1.08) |  |
| 14 | 738 | 6.51 | 256 | | 7.15 | 0.91 (**0.81**, 1.02) |  |
| 18C | 740 | 2.08 | 255 | | 2.26 | 0.92 (**0.84**, 1.02) |  |
| 19A | 739 | 4.71 | 255 | | 4.88 | 0.97 (**0.87**, 1.07) |  |
| 19F | 740 | 4.19 | 256 | | 4.37 | 0.96 (**0.87**, 1.06) |  |
| 23F | 701 | 2.18 | 240 | | 2.30 | 0.95 (**0.85**, 1.06) |  |
| CI, confidence interval; N, number of children with pre-and post-vaccination results available. Adjusted GMC, geometric mean antibody concentration adjusted for pre-vaccination concentration. PCV13 subset: subset of children in the VZV subset for whom we tested immunogenicity of the 13-valent pneumococcal conjugate vaccine. See details in Supplementary material. ^a^ 95% confidence interval calculated with an analysis of covariance (ANCOVA) model with pre-vaccination titer as regressor. Values in bold indicate non-inferiority criterion met: lower limit of the two-sided 95% CI for the adjusted GMC ratio at Day 42 (MMR-RIT over MMR II) ≥0.5. | | | | | | | |

**Supplementary Table 5.** Incidence of rash and febrile convulsions or signs of meningeal irritation (Day 0–42) (total vaccinated cohort).

| **n (%)** | **MMR-RIT  (N=3566)** | **MMR**  **II (N=1243)** |  |
| --- | --- | --- | --- |
| Rash (localized or generalized) | | | |
| Any rash | 1043 (29.2) | 378 (30.4) |  |
| Related | 489 (13.7) | 162 (13.0) |  |
| Grade 3 | 106 (3.0) | 25 (2.0) |  |
| Measles/rubella-like | 235 (6.6) | 77 (6.2) |  |
| Varicella-like | 250 (7.0) | 85 (6.8) |  |
| Febrile convulsions or signs of meningeal irritation | | |  |
| Any | 10 (0.3) | 3 (0.2) |  |
| Related | 4 (0.1) | 2 (0.2) |  |
| Grade 3 | 4 (0.1) | 0 (0.0) |  |
| N, number of children with the documented dose; n (%), number (percentage) of children reporting the adverse event at least once. Related: adverse event assessed by the investigator as causally related to study vaccination. Grade 3 febrile convulsions or rashes were those preventing normal, everyday activities. | | |  |

**Supplementary Table 6.** Incidence of unsolicited adverse events (Day 0–42), serious adverse events, AEs prompting emergency room visits and NOCDs (Day 0–180) (total vaccinated cohort).

| **n (%)** | **MMR-RIT (N=3714)** | **MMR II (N=1289)** |
| --- | --- | --- |
| Unsolicited AEs (≥1 symptom) | 1857 (50.0) | 618 (47.9) |
| Grade 3 | 225 (6.1) | 85 (6.6) |
| SAEs (any, ≥1 SAE) | 77 (2.1) | 25 (1.9) |
| AEs prompting ER visit | 375 (10.1) | 134 (10.4) |
| NOCDs (any, ≥1 NOCD) | 128 (3.4) | 48 (3.7) |
| AE, adverse event; ER, emergency room; N, number of children with the documented dose; n (%), number (percentage) of children reporting the AE at least once; NOCDs, new onset chronic diseases (see definition in Patients and methods); SAE, serious AE. Grade 3 unsolicited AEs were those preventing normal, everyday activities. | | |

**Supplementary Figure 1.** Study design.

**
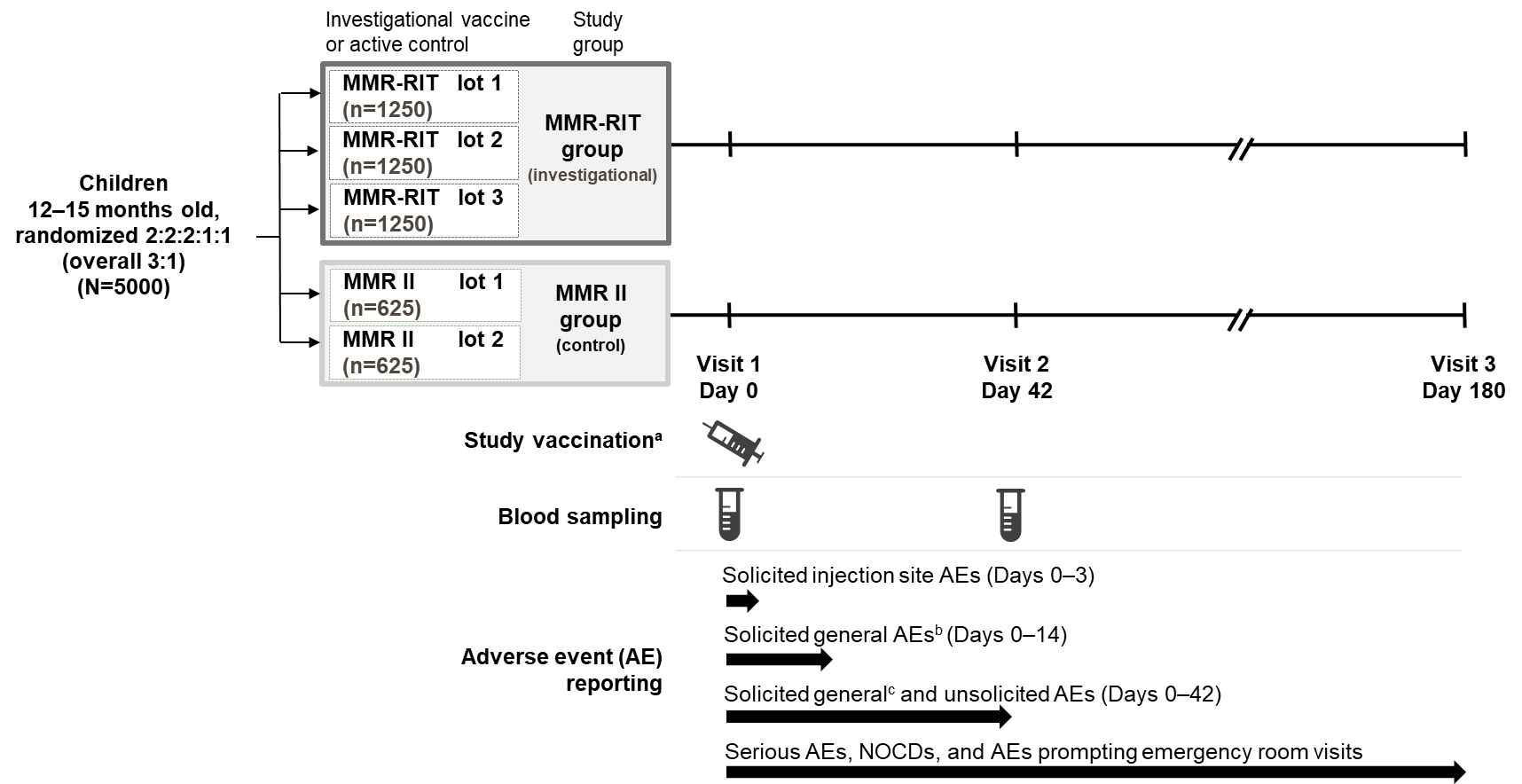
**

N, planned number of enrolled children; n, planned number of enrolled children in each study group; NOCDs, new onset chronic diseases. ^a^Study vaccines were co-administered with varicella and hepatitis A vaccines in all children, and with the 13-valent pneumococcal conjugate vaccine in children in the US. ^b^Drowsiness, loss of appetite and irritability/fussiness. ^c^Fever, rash, parotid/salivary gland swelling, and febrile convulsions or signs of meningeal irritation.

**Supplementary Figure 2.** Incidence of solicited injection site adverse events (Day 0–3), fever (Day 5–12), and drowsiness and loss of appetite (Day 0–3) (total vaccinated cohort).

**
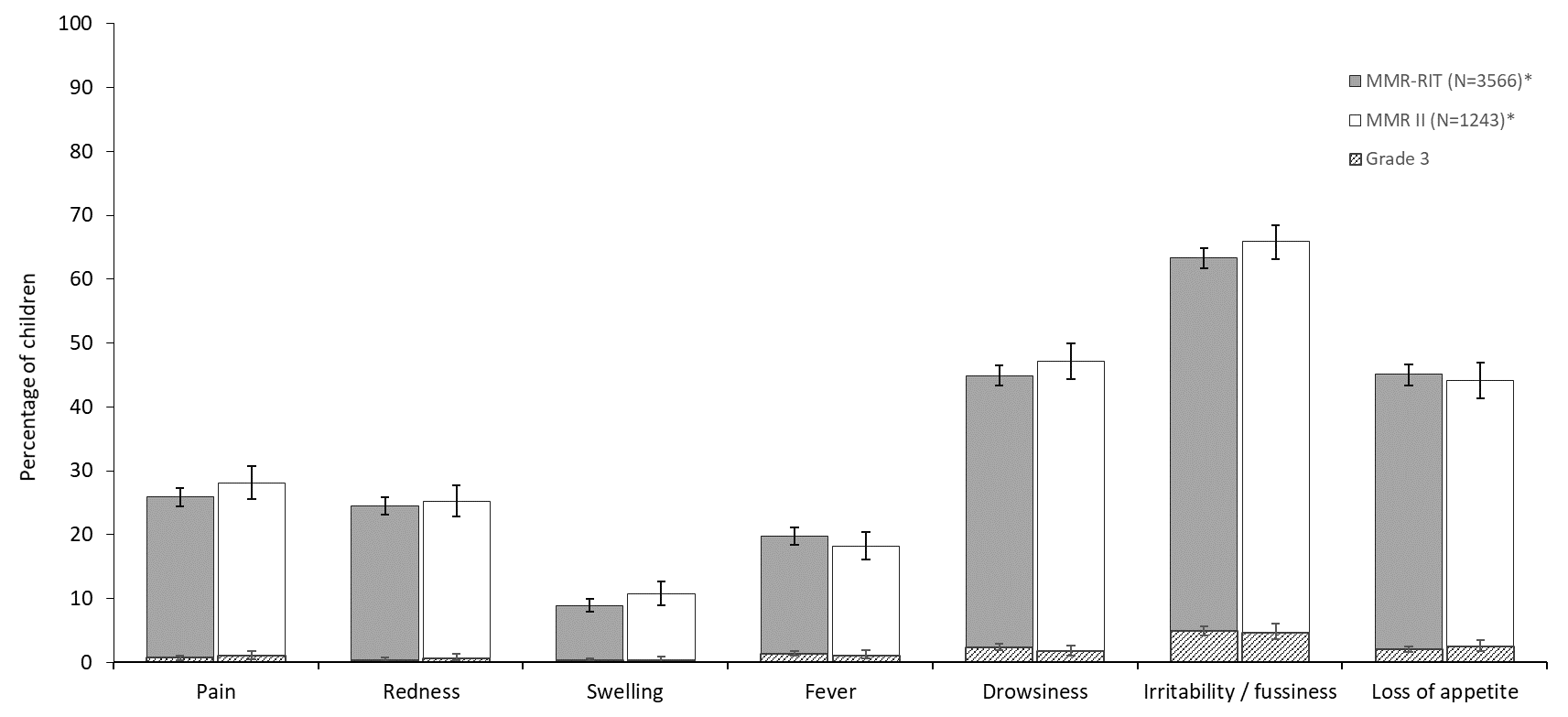
**

N, number of children with the documented dose. *Except for pain, redness, and swelling (MMR-RIT, N=3555 and MMR II, N=1242).
The injection site adverse events (i.e., pain, redness, and swelling) refer to the site of MMR vaccine injection. Fever: temperature ≥38.0°C. Grade 3 was defined as: limb spontaneously painful or child cried when limb was moved (pain); diameter >20 mm (redness, swelling); temperature >39.5°C (fever); adverse event preventing normal, everyday activities (drowsiness); not eating at all (loss of appetite); crying inconsolably (irritability/fussiness). The error bars represent the upper and lower limits of the exact two-sided 95% confidence intervals.
